# Viewers Change Eye-Blink Rate by Predicting Narrative Content

**DOI:** 10.3390/brainsci11040422

**Published:** 2021-03-26

**Authors:** Celia Andreu-Sánchez, Miguel Ángel Martín-Pascual, Agnès Gruart, José María Delgado-García

**Affiliations:** 1 Neuro-Com Research Group, Department of Audiovisual Communication and Advertising, Universitat Autònoma de Barcelona, 08193 Barcelona, Spain; miguelangel.martin@rtve.es; 2 Innovation and Technology, Instituto de Radio Televisión Española, Corporación Radio Televisión Española, 08174 Sant Cugat del Vallès, Spain; 3 Division of Neurosciences, Pablo de Olavide University, 41013 Sevilla, Spain; agrumas@upo.es (A.G.); jmdelgar@upo.es (J.M.D.-G.)

**Keywords:** visual perception, attention, cognitive neuroscience, media content, neurocinematics

## Abstract

Eye blinks provoke a loss of visual information. However, we are not constantly making conscious decisions about the appropriate moment to blink. The presence or absence of eye blinks also denotes levels of attention. We presented three movies with the exact same narrative but different styles of editing and recorded participants’ eye blinks. We found that moments of increased or decreased eye blinks by viewers coincided with the same content in the different movie styles. The moments of increased eye blinks corresponded to those when the actor leaves the scene and when the movie repeats the same action for a while. The moments of decreased eye blinks corresponded to actions where visual information was crucial to proper understanding of the scene presented. According to these results, viewers’ attention is more related to narrative content than to the style of editing when watching movies.

## 1. Introduction

While watching media content, there is something we do constantly that we hardly notice but that reflects our attention: blinking. On average, humans blink between eight and 21 times per minute while resting [1], but our eye-blink rate changes when we carry out different activities such as talking, listening, looking around, or watching screens. Eye blinks have the primary physiological function of wetting the cornea [2,3]. They also hide visual flow for a short (100–400 ms) period of time [4,5]. As a result, on average, an adult spends ~44 min/day awake but with their eyelids closed [6]. During that period, visual information is not perceived. In accordance, we have to decide, whether consciously or not, the best moment to blink in order to lose the least possible amount of visual information.

There remains a lot to study about the neurobiological basis of eye blinks. For example, it has been suggested that the spinal trigeminal complex is an integral component of the spontaneous eye-blink generator circuit [7]. It has also been suggested that the basal level of corneal afferent input to the spinal trigeminal complex establishes the interblink interval [7]. Spontaneous eye blinks involve a dynamic alteration of brain activity, with a prominent but momentary activation of the bilateral hippocampus and the cerebellum after the blink onset, when subjects view videos attentively [8]. Also, it has been suggested that the eye-blink rate can be used as a noninvasive indirect marker of central dopamine function, with a higher eye-blink rate predicting higher dopamine function [9]. Blink rate has also been associated with enhanced learning from negative outcomes, helping to suggest that lower dopamine levels per se may improve learning from negative choices [10]. A recent study suggested that blink modulation is related to the motivational and biological significance of the stimuli, providing a solid background for the study of emotion–attention patterns using a noninterfering psychophysiological measurement [11].

The blink rate has been linked to attention in several circumstances and varies according to mental activities. Blinks are perceived as communicative signals in human face-to-face interaction, directly influence speakers’ communicative behavior in this context [12] and can be used to distinguish liars from truth-tellers [13]. An increase of the eye-blink rate has been correlated with a decrease of attention, and vice versa [14,15,16]. Compared with a control state, the blink rate is higher during face-to-face conversation but decreases during a classroom examination [17]. Blinks also play an important role in the perception of magic shows [18]. Stemming from these many investigations, eye blinks are used as attentional markers. In the media context, it is known that attention is one of the most important variables to consider when creating a movie, and there are several ways to manage elements in designing a visual work that is attractive to viewers. Two of these elements are content and style.

### 1.1. Content: Storytelling and Attention

The narrative is the content and the way it is explained to spectators. Both for watching media content and for creating the visual meaning of the world around us, one of the most central internal functions is attention [19]. Since the dawn of media productions, communication researchers have adopted various approaches to determine how to quantify and manage attention in narratives [20,21,22]. Recent studies have demonstrated that one way to determine viewers’ attention to media content is through a proper quantification of involuntary or spontaneous eye blinks [23,24,25,26]. Eye-blink patterns are certainly linked to communication processes. While viewers are watching and listening to a speaker, their eye blinks are synchronized with the speaker’s blinks, with a delay of around 100 ms [27]. However, such entrainment does not occur when viewers watch speech without sound or listen to the sound of speech without video stimulus. This indicates that blink entrainment is not an automatic imitation of an observed behavior but rather a reflection of narrative comprehension [27]. Blinking has also been linked to autism spectrum disorder (ASD), and it is of interest in the context of linking attention to blinks. While adults without ASD significantly synchronize their eye blinks to those of speakers, ASD listeners do not [28], and non-ASD toddlers inhibit their blinking earlier than toddlers with ASD, thus maximizing access to visual information and anticipating forthcoming events [6]. Based on these results, it has been proposed that measurements of blink inhibition can provide an index of autonomic reactivity and differential engagement, and of the viewer’s subjective assessment of the importance of media content. By measuring the timing of blink inhibition relative to content, one can determine the viewer’s subjective assessment of the importance of what he or she is watching [6] and, thus, confirm how story-telling affects viewers’ attention.

### 1.2. Style: Media and Attention

Editing is part of the style and the way information is presented visually, consisting of the fragmentation of the content within shots and camera movements. Editing has been studied since the early work by Griffith in the 1910s [29,30] and the Soviet school of cinema. In the latter case, editing was analyzed on the basis of different experiments and theories, such as those of Kuleshov [31], Pudovkin [32], and Eisenstein [33]. Editing is part of the cinematographic language, and media creators use it as a unifying mechanism of projection and identification, and to construct realistic impressions [34]. Despite the interest in creating realistic scenes in media works, real scenes and those in media are perceived differently by spectators. Media content inhibits viewers’ eye-blink rate significantly compared with the same narrative in reality [26]. In addition, recent investigations have proven that the style of editing affects viewers’ attentional level as indicated by their eye-blink rate [24]. The more chaotic and discontinuous the editing style, the lower the eye-blink rate of viewers and, presumably, the greater their attention. Cuts inhibit viewers’ eye blinks, thus media creators can use them to manage attention [25]. One reason why viewers tend, unconsciously, to avoid blinking when cuts are inserted into media content could be that the film whose visual flow is interrupted by the cut “blinked” for them; another explanation is that, when the image changes, viewers avoid losing visual information by inhibiting eye blinks. Cuts break up familiar contexts since they present new visual information to be decoded. The lack of familiarity becomes a lack of prediction, thus decreasing the efficiency of sensory (visual, auditory) perception [35].

Cuts also affect viewers’ attention and brain activities, and event boundaries in narrative movies provoke transient brain responses [36]. Event boundaries represent different types of changes in films. Among others, event boundaries include spatial, temporal, object, or character changes, being associated with increased segmentation into parts by viewers for the sake of the proper recognition of objects [37]. The perception of event boundaries would be a side effect of prediction during ongoing perception. Interestingly, cuts are not associated with increases in subjective perception of segmentation [38]. The predictability of incoming information influences event perception and thereby narrative comprehension. However, since cuts are not by themselves associated with increases in event segmentation, it may be understood that formal categories used to classify cuts are predictive of event segmentation [39]. Moreover, the phenomenon of edit blindness means that film viewers are unaware of some film edits [40]. Many film editors and researchers assume that editing in accordance with so-called continuity editing rules favors viewers’ edit blindness [40]. This idea suggests that differences could be found in the attention of viewers watching continuous and discontinuous editing styles, and thus, that media editing is highly related to attention.

### 1.3. Synchronization in Media Perception

In communication contexts, learning about patterns and synchronization is of great interest to media creators. In the 1990s, Walter Murch, a Hollywood film editor, wondered whether there is an optimal moment to insert a cut during the editing process to respect eye blinks and thus avoid the loss of visual information [41]. Murch, who worked on films such as *The Godfather* (1972) and *Apocalypse Now* (1979), suspected that eye blinks could have a comprehension function in the proper understanding of a movie.

During the last few decades, the synchronization in viewers’ perception has been proven through the inter-subject correlation model [42,43]. A highly significant tendency for the brains of different individuals to act in unison during free viewing of movie scenes has been reported [42]. Such inter-subject synchronization has been correlated with emotionally arousing scenes [42]. Synchronization of eye blinks while viewing video stories has also been reported [23]. In their investigation, Nakano and colleagues found that the synchronization of eye blinks occurred during scenes in which the narrative required less attention, such as the conclusion of an action, the absence of the main character, or during a repetition of the same scene or the presentation of a similar one. Synchronized blinks have also been found in the perception of magic shows, where such synchronization of blinks between spectators occurs after a seemingly impossible feat [18]. All these results lead us to consider that there may be patterns in media perception and that viewers may control, consciously or not, the timing of their responses, such as blinking, to avoid the loss of important visual information.

Since we know that perception in a media context is synchronized at some points, we wondered what would govern such synchronization: content or style. To investigate this, in this study we compared the eye blinks of viewers while they watched three different movies with the same content but different styles of editing. The aim was to check whether the content of the movie, regardless of the style, can control viewers’ eye-blink rate, or, on the contrary, the style governs the perception, regardless of the content.

## 2. Materials and Methods

### 2.1. Participants

Forty human subjects (age 43.97 ± 8.07 years) with normal or corrected-to-normal vision participated in the experiment. Participants gave prior written informed consent to participate in the study. The experiment was carried out in accordance with relevant guidelines and regulations for human research and was approved by the Ethics Commission for Research with Animals and Humans (CEEAH) of the Universitat Autònoma de Barcelona (Barcelona, Spain).

### 2.2. Stimuli

We created three video stimuli, each of 198 s duration, with the same narrative and content, but different editing styles. One stimulus was a one-shot movie consisting of a single open shot with no cuts. The second video stimulus was a movie with 33 shots and a continuous, classic style of editing, with an average shot length of 5.9 s. This stimulus presented classic shots with smooth transitions in accordance with the 180° rule, by which the same action is filmed following that angle to avoid spatial discontinuity. The third video stimulus was a movie with 79 shots and a discontinuous, chaotic style of editing, with an average shot length of 2.4 s. This third stimulus broke the classic 180° rule, and presented sudden movements in the frame, discontinuities in time and space between shots, constant camera movements, and a large number of different kinds of shots.

The narrative consisted of a man who enters a room containing a desk, goes out, enters again, and sits at the desk. On the desk there are three colored balls, three books, and an apple. He juggles with the colored balls, puts them back on the desk, and goes out again. The man enters once more with a laptop in a case, sits, opens the case, and takes out the device. He opens it up and works on it, then picks up, one by one, each of the three books on a desk situated on the right side of the screen, reads something in it, then puts it down on the left side of the screen. He works for a while with the laptop, then closes it and moves it to the left. Then, the man puts his hand into his pocket and takes out a small torch, which he points towards the viewer. He turns it on for a few seconds, turns it off, then puts it back into his pocket. He takes the apple from the desk, rubs it on his shoulder, and bites it. He chews and bites the apple repeatedly for a while. He leaves the apple core behind the laptop, to the left of the screen. The man swallows the rest of the apple and wipes his mouth with his hand. Then he makes a happy face, a sad face, and a disgusted face, runs his hand over his face, and makes a happy face again. The man stands up and leaves the room.

Stimuli were presented on a high-definition (HD) 42-inch light-emitting diode (LED) display (TH42PZ70EA, Panasonic Corporation, Osaka, Japan) using Paradigm Stimulus Presentation software (Perception Research System Incorporated, Laurence, KS, USA).

### 2.3. Data Acquisition

Subjects participated in sessions (~15 min) of active viewing. All participants watched all the stimuli. The presentation of the stimuli was randomized over all possible combinations. The stimuli were presented on a stage that we designed to make participants feel comfortable while watching the media content. We asked participants to watch the stimuli without further requirements, having told them that they would be asked some questions after the visualization. At the end of the session, participants filled out a distracting questionnaire.

Observers’ eye blinks were detected following a dual protocol: using electroencephalographic/electromyographic (EEG/EMG) recordings and a HD video recording system. Participants’ EEG/EMG was recorded using a wireless device (Enobio^®^, Neuroelectrics, Barcelona, Spain) with 20 electrodes placed according to the 10–20 International System. Eye blinks were detected by the prefrontal Fp1 and Fp2 electrodes and electrooculographic electrodes. For comparison, participants’ faces were also recorded in a close-up shot with an HD camera (HDR-GW55VE, Sony Corporation, Tokyo, Japan) at 25 frames/s.

### 2.4. Data Analysis

We analyzed eye blinks following two procedures. Firstly, we filtered EEG/EMG data to 0.5–3 Hz and applied Brainstorm’s eye-blink detectors (Brainstorm, University of Southern California, Los Angeles, CA, USA) in electrooculographic (EOG), Fp1 and Fp2 channels, running on MATLAB R2013a (The Mathworks Inc., Natick, MA,). In a second step, we manually checked eye blinks in the videos of participants’ faces recorded with the HD camera. Using those two methods, we obtained a matrix with a final list of each participant’s eye blinks. To assess changes in blink rate with time, each video was divided into 40 blocks of 4.95 s, and the blink number was converted into blinks/min for each block. The blink rate analysis was performed by repeated-measures analysis of variance (ANOVA) designed with two factors: time and style of editing. The time factor corresponds with each of the blocks. Using the blinks within each block, we computed a two-way ANOVA with blocks that showed increases or decreases, and the rest of them, with type of block and style of editing as factors. We used SigmaPlot 11.0 (Systat Software Inc., San Jose, CA, USA) for the statistical analysis.

## 3. Results

**Figure 1:**
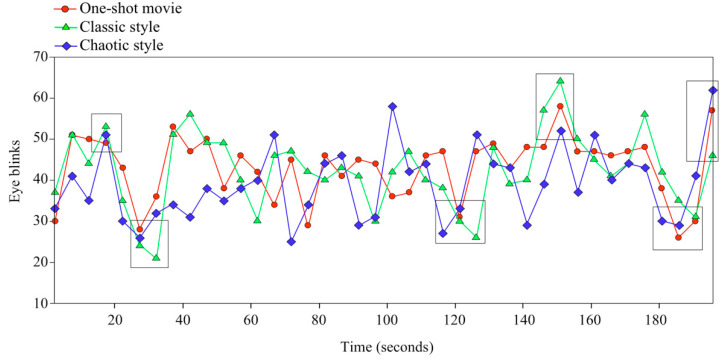
Timeline of the video, showing histograms of eye blinks from all participants (*N* = 40) while watching the movie with each style. Red circles indicate blinks during the one-shot movie; green triangles those during the continuous movie, edited with a classical style; and blue squares those during the movie with discontinuous and chaotic style. The distribution is into 40 bins of 4.95 s each. The boxes indicate the moments and actions when viewers’ eye blinks coincide among the different styles of editing.

We detected a total of 1721 blinks in the one-shot movie, 1688 blinks in the classical-style movie, and 1560 blinks in the chaotic-style movie. We distributed all the eye blinks into 40 bins of 4.95 s each for presentation as a histogram for further comparisons and analyses (Figure 1). We observed that the evolution of the eye blinks through those bins was very similar, regardless of the style of editing presented to participants. This result made us suspect that movie content would be more relevant than style in the distribution of eye blinks across time. The blink rate analysis revealed a significant main effect of Time (*F*_(39,3041)_ = 5.199, *p* < 0.001) and a significant Time × Style interaction (*F*_(78,3041)_ = 2.004, *p* < 0.001). No significant main effect of Style was found (*F*_(2,3041)_ = 2.982, *p* = 0.057).

**Figure 2:**
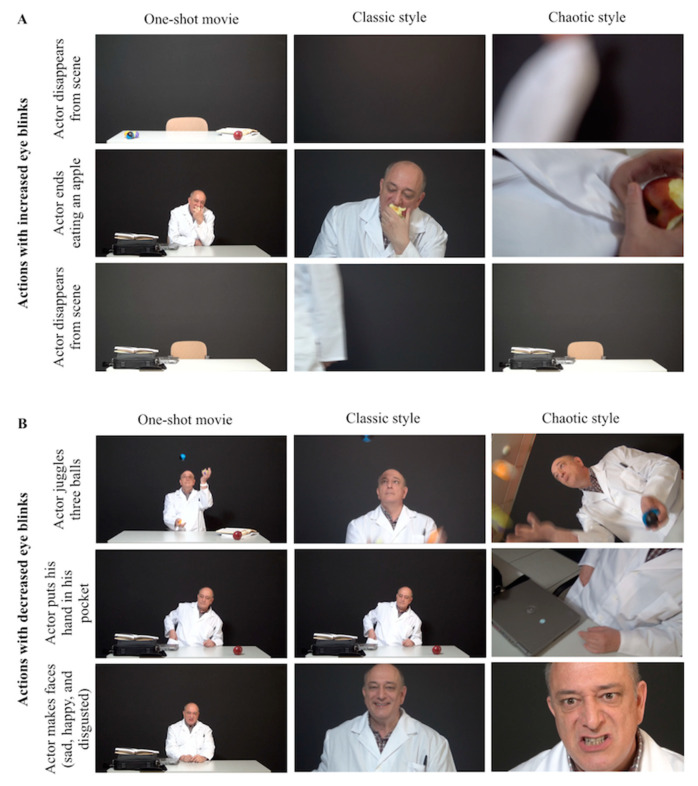
Moments with increased and decreased eye blinks while watching the same narrative with three different styles of editing. (**A**) The three moments when increased eye blinks were observed regardless of the style of editing (from top to bottom): when the actor disappears from the scene near the beginning of the movie, when the actor finishes eating the apple, and when the actor disappears from the scene at the end of the movie. (**B**) The three moments when decreased eye blinks were observed regardless of the style of editing (from top to bottom): when the actor juggles three balls, when the actor puts his hand in his pocket, and when the actor makes faces (sad, happy, and disgusted). The person appearing in Figure 2 is co-author Miguel Ángel Martín-Pascual. Dr. Martín-Pascual consents to the appearance of his image in this publication.

According to these results, there were six actions in the narrative content when special coincidence between the participants’ eye blinks was observed for all three stimuli presented: three corresponding to increased and the others to decreased eye blinks (Figure 1). Since these coincidences were found even when the style of the video was different, we looked at the narrative occurring at these precise moments. The actions with increased eye blinks were the following (Figure 2A): when the actor disappeared from the scene (at around second 20), when the actor finished eating the apple (at around second 150), and at the end of the video, when the actor left the scene again (at around second 192). At the moments where the viewers’ eye blinks decreased, we found the following actions (Figure 2B): the actor is juggling (at seconds 20–30), the actor puts his hand into his pocket (at around second 120), and the actor is making happy, sad, and disgusted faces (at seconds 180–190).

The mean (±SEM) number of eye blinks in the blocks with an increase corresponding to the mentioned actions was 55.89 (±2.18), compared with 29.14 (±1.72) for the bins with a decrease, while for the rest of the bins without an increase or decrease it was 42.03 (±0.67). We compared the mean number of eye blinks during the moments (or blocks) where there was a coincident increase, a coincident decrease, and the rest, also considering the style of editing. A descriptive analysis based on the style of editing showed that the mean (±SEM) number of blinks in the blocks with an increase was 54.67 (±3.78) for the one-shot movie, 58 (±3.78) for the movie with the classical, continuous style of editing, and 55 (±3.78) for the movie with a chaotic style of editing. For the bins with decreased eye blinks, the mean was 28.75 (±3.28) for the one-shot movie, 29.66 (±2.67) for the movie with the continuous style of editing, and 29 (±2.93) for that with a chaotic style of editing. For the rest of the bins, the mean was 43.76 (±1.14) for the one-shot movie, 43.16 (±1.18) for the movie with a continuous style of editing, and 39.16 (±1.16) for that with a chaotic style of editing. A two-way ANOVA showed that the moments (Time) affected viewers’ eye blinks significantly (*F*_(2,119)_ = 47.963, *p* < 0.001). There was not a statistically significant interaction between Style and Time (*F*_(4,119)_ = 0.472, *p* = 0.76). We then carried out post hoc multiple-comparison procedures (Holm–Šídák method), with an overall significance level of *p* < 0.05. We found that, while the style of editing was significant within blocks showing no increase or decrease (*p* < 0.05), it was not significant within the blocks showing an increase (*p* > 0.05) or decrease (*p* > 0.05) of viewers’ eye blinks. These results confirm that the style of editing [17] is not the only thing that governs the increase or decrease of participants’ eye blinks while watching media content.

## 4. Discussion

We had previously found that the style of editing affects viewers’ eye-blink rate, especially the chaotic style [24]. Here, we found that synchronization between increased and decreased viewers’ eye blinks occurred at some specific moments of the timeline while they watched a movie, regardless of the style of editing of the movie but linked to its content. This agrees with the idea that, when watching media content, blinks are generated, in part, because of cognitive processing related to the narrative [23]. This study suggests that content can be used as a specific procedure to manage viewers’ attention independently of the style of editing. Previous studies had already proven that different techniques can relax the audience’s attention depending on the content, such as those used by magicians to perform their tricks [18]. However, no study had compared how different styles of editing would affect viewers’ attention to the same content. According to our results, the coincidence of viewers’ attention is more related to the content and narrative than to the style.

Two of the three moments when participants increased their eye blinks occurred in the absence of the actor. This is coincident with the findings of a previous study [23]. There was a third moment when increased eye blinks occurred with the actor still in the scene. At this third moment, the actor had been performing the same action (eating an apple) for 20–30 s. These results are in accordance with a previous investigation that found synchronization in viewers’ eye blinks during repetition of the same scene [23]. In accordance with those findings, we propose that such increases of eye blinks may be related to the prediction ability of viewers. They have been seeing the same action for a long while and can easily predict what is going to happen since the action has not changed much. If something is predictable, it needs less attention. This would relate to Hawkins’ theory of the memory–prediction framework. According to Hawkins, prediction is a tool that is commonly used when knowledge of past events can be applied to new situations that are similar to the past [44]. Our results suggest that this may be the reason why viewers increased their eye blinks: to take advantage of the opportunity to blink when they already know what is happening in the scene.

It is of interest to point out that, although repetition of an action or substantial knowledge of what is going to happen during media content has been linked to increased eye blinks and thus a probable decrease of attention, musical videoclips that are continually replayed on video-sharing platforms become phenomena that capture audiences’ attention. On the other hand, we have previously found that media professionals (who are used to constantly watching audiovisuals and thus expected to have greater expertise and ability to predict audiovisual content) exhibit significantly decreased eye-blink rates compared with people who are not media professionals while watching audiovisual content [45]. Further investigations should explain these apparent contradictions.

The three moments with decreased eye blinks have in common the need for alertness to what might happen next. First, in the juggling part, it is understood that viewers do not want to miss information of where the balls are at each moment. That action entails the risk that the balls will fall at any time. Second, in the pocket part, the viewers seem to need to pay attention to what the actor might take out. Again, there is a risk of the unknown: “what could be revealed?” Third, in the faces part, viewers might decrease their eye-blink rate to avoid loss of information and to identify the actor’s emotions [46]. Such decreases of eye blinks at moments when the action seems to be unpredictable may be an unconscious strategy to avoid loss of important visual information.

## 5. Conclusions

A previous study reported that cuts significantly inhibit viewers’ eye blinks [36]. However, according to the present results, viewer’s eye blinks are more related to content than to style when watching movies. Spontaneous nonconscious eye blinks have been linked to the default mode network [47], which is known to counteract the dorsal attention network and which is involved in introspection [48,49]. Our results suggest that content can be used to increase or decrease spontaneous eye blinks. Thus, we suggest that, in the context of managing viewers’ attention, content overrules style. Media creators can use this finding to enhance viewers’ attentiveness. The interesting output of this investigation is that it seems possible to create patterns (such as the increase of viewers’ eye-blink rate during the disappearance of the main actor from the scene) that would be useful for script writers and media producers. Further research should assess common actions and situations in audiovisual content to identify more patterns in viewers’ attention.

## Data Availability

The data presented in this study are available in Table S1.

## Supplementary Materials

The following are available online at https://www.mdpi.com/article/10.3390/brainsci11040422/s1, Table S1: Distribution of eye blinks/min in 40 blocks of 4.95 s each.
